# Loss-of-function mutation of c-Ret causes cerebellar hypoplasia in mice with Hirschsprung disease and Down's syndrome

**DOI:** 10.1016/j.jbc.2021.100389

**Published:** 2021-02-06

**Authors:** Nobutaka Ohgami, Akira Iizuka, Hirokazu Hirai, Ichiro Yajima, Machiko Iida, Atsuyoshi Shimada, Toyonori Tsuzuki, Mayumi Jijiwa, Naoya Asai, Masahide Takahashi, Masashi Kato

**Affiliations:** 1 Department of Occupational and Environmental Health, Nagoya University Graduate School of Medicine, Nagoya, Aichi, Japan; 2 Unit of Environmental Health Sciences, Department of Biomedical Sciences, College of Life and Health Sciences, Chubu University, Kasugai, Aichi, Japan; 3 Department of Neurophysiology and Neural Repair, Gunma University Graduate School of Medicine, Maebashi, Gunma, Japan; 4 Pathology Research Team, Faculty of Health Sciences, Kyorin University, Mitaka, Tokyo, Japan; 5 Department of Surgical Pathology, Aichi Medical University Hospital, Nagakute, Aichi, Japan; 6 Department of Pathology, Nagoya University Graduate School of Medicine, Nagoya, Aichi, Japan; 7 Department of Pathology, Fujita Health University, Toyoake, Aichi, Japan; 8 International Center for Cell and Gene Therapy, Fujita Health University, Toyoake, Aichi, Japan

**Keywords:** receptor tyrosine kinase, phosphotyrosine, cerebellum, neurological disease, neurite outgrowth, sonic hedgehog (Shh), DS, Down's syndrome, EDNRB, endothelin receptor B, EGL, external germinal layer, ENS, enteric nervous system, GCs, granule cells, GCPs, granule neuron progenitors, GDNF, glial cell line–derived neurotrophic factor, GFRα1, GDNF family receptor α1, HSCR, Hirschsprung disease, IGL, internal granular layer, KI, knock-in, PCs, Purkinje cells, Shh, sonic hedgehog

## Abstract

The c-*RET* proto-oncogene encodes a receptor-tyrosine kinase. Loss-of-function mutations of *RET* have been shown to be associated with Hirschsprung disease and Down's syndrome (HSCR-DS) in humans. DS is known to involve cerebellar hypoplasia, which is characterized by reduced cerebellar size. Despite the fact that c-Ret has been shown to be associated with HSCR-DS in humans and to be expressed in Purkinje cells (PCs) in experimental animals, there is limited information about the role of activity of c-Ret/c-RET kinase in cerebellar hypoplasia. We found that a loss-of-function mutation of c-Ret Y1062 in PCs causes cerebellar hypoplasia in *c-Ret* mutant mice. Wild-type mice had increased phosphorylation of c-Ret in PCs during postnatal development, while *c-Ret* mutant mice had postnatal hypoplasia of the cerebellum with immature neurite outgrowth in PCs and granule cells (GCs). *c-Ret* mutant mice also showed decreased numbers of glial fibers and mitogenic sonic hedgehog (Shh)-positive vesicles in the external germinal layer of PCs. c-Ret-mediated cerebellar hypoplasia was rescued by subcutaneous injection of a smoothened agonist (SAG) as well as by reduced expression of *Patched1*, a negative regulator for Shh. Our results suggest that the loss-of-function mutation of c-Ret Y1062 results in the development of cerebellar hypoplasia *via* impairment of the Shh-mediated development of GCs and glial fibers in mice with HSCR-DS.

The c-*RET* proto-oncogene is one of the receptor-tyrosine kinases. c-RET is known as one of the receptors for a glial cell line–derived neurotrophic factor (GDNF) (1). The GDNF acts on target cells by binding to a GDNF family receptor α1 (GFRα1), which then leads to the formation of a signaling complex with c-RET. The formation of this complex leads to autophosphorylation in c-RET, resulting in activation of c-RET-mediated intracellular signaling pathways (1, 2, 3, 4). c-Ret is known to have an intracellular kinase domain with specific tyrosine residues including tyrosine 1062 (Y1062), which serves as not only a crucial autophosphorylation site for its kinase activation but also a multidocking site for several signaling pathways (5, 6, 7). *c-Ret*/c-*RET* has been shown to play important roles for the development of the enteric nervous system (ENS) and the kidney in humans and experimental animals (5, 6, 8).

The cerebellum, which is required for coordinate performance, consists of layered structures with several cell types. The external germinal layer (EGL) is located in the outermost layer containing dividing granule cell progenitors (GCPs). After mitosis, granule cells (GCs) inwardly migrate along glial fibers of Bergmann glia from the EGL and then penetrate through the layer of Purkinje cells (PCs) to form the internal granular layer (IGL), where GCs eventually maturate (9). Proliferation and differentiation of GCPs continue and the thickness of the EGL gradually decreases until ∼3 weeks of age after birth (9). Analyses of cerebellar granule cell development have shown that proliferation and migration of GCPs require mutual communication to PCs and can be facilitated by several mitogenic factors (9, 10). A previous study showed that c-Ret was present in PCs but not in GCs and that phosphorylation of c-Ret kinase occurred in the cerebellum of the rat after birth (11), raising the possibility that c-Ret kinase is linked to postnatal development of the cerebellum. At present, however, there is limited evidence indicating a correlation between phosphorylation of *c-Ret/c-RET* and postnatal development of the cerebellum.

Hirschsprung disease (HSCR), which affects one in 5000 births, is a congenital disorder of the ENS with an aganglionic megacolon, impaired development of the kidneys, and deafness. Most cases are thought to be multigenic and multifactorial (12, 13). In previous studies, HSCR has been shown to involve Down's syndrome (HSCR-DS) (14). DS is known to involve cerebellar hypoplasia in addition to impaired development of the ENS and hearing loss (15). In a previous study, a significant association of endothelin receptor B (*EDNRB*) with HSCR-DS was shown in humans (16). An experimental study demonstrated impaired development of GCs in the EGL in the cerebellum in rats with mutation of *Ednrb* (17). In this study, impaired coordinate performance was also shown in *Ednrb*-knockout (−/−) mice (Fig. S1), suggesting that cerebellar hypoplasia develops in *Ednrb*-knock-out (−/−) mice with HSCR including deafness (18). On the other hand, a small-scale study showed that 14 patients diagnosed with DS-associated HSCR had mutations in *c*-*RET*, suggesting the possibility that *c-RET* is one of the major causal genes for HSCR-DS (14). *c-Ret* homozygous knock-in mice, in which Y1062 in c-Ret was replaced with phenylalanine (*c-Ret*-KI^YF/YF^-mice), have been shown to develop HSCR including severe impairments of the ENS and kidney (3) and congenital hearing loss (19). However, there is no evidence that *c-Ret*-KI^YF/YF^-mice involve cerebellar hypoplasia, which is included in HSCR-DS.

The aim of this study was to determine whether cerebellar hypoplasia occurs in *c-Ret*-KI^YF/YF^-mice with HSCR. Our results demonstrated for the first time that impaired phosphorylation of c-Ret in PCs causes severe cerebellar hypoplasia in *c-Ret*-KI^YF/YF^-mice with impaired development of GCs.

## Results

### Increased phosphorylation of c-Ret Y1062 in Purkinje cells during postnatal development and motor impairments in c-Ret-KI^YF/YF^-mice

**Figure 1:**
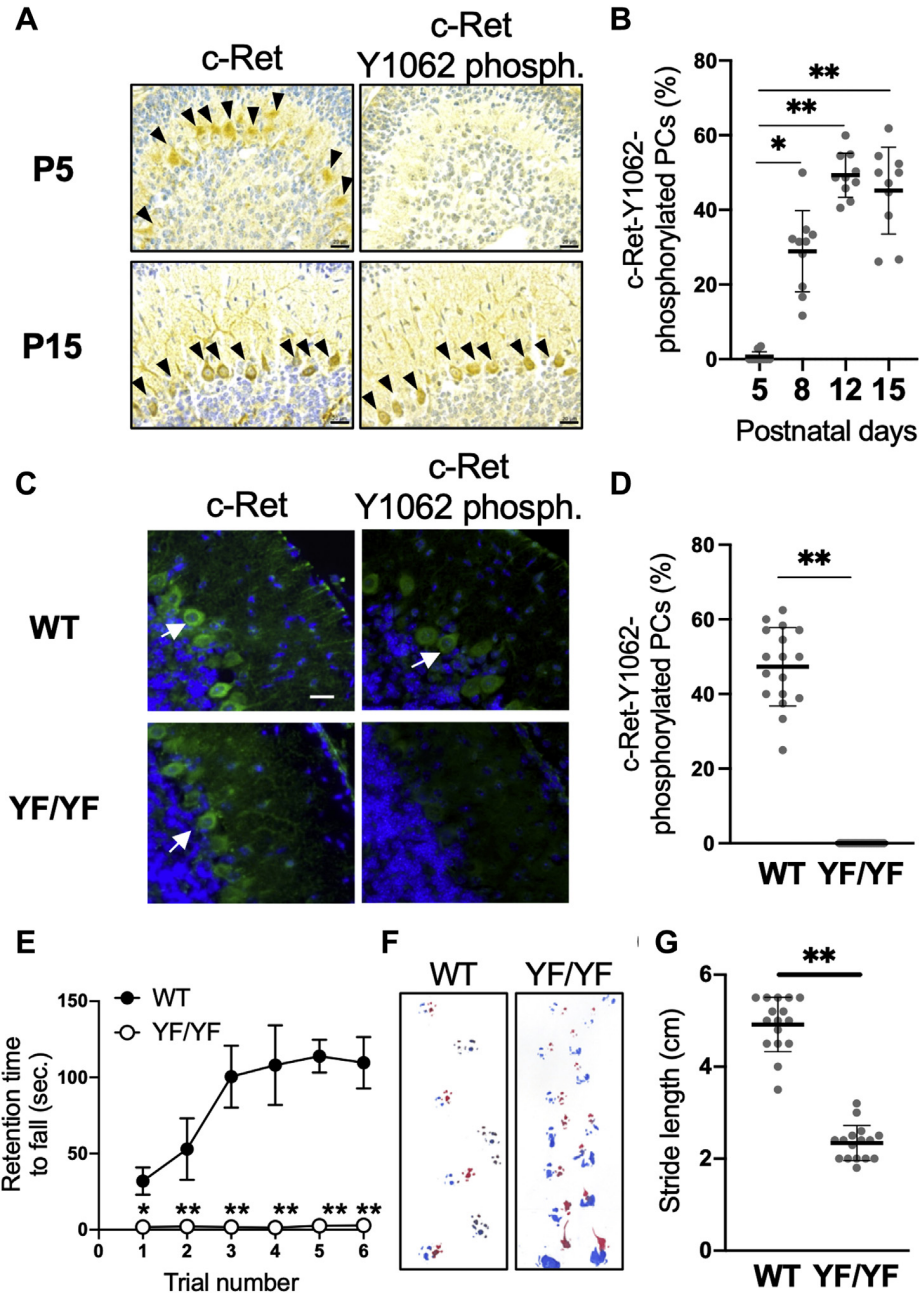
**Postnatal increase of c-Ret Y1062-phosphorylated PCs.***A* and *B*, c-Ret-expressing (*arrowheads* in *upper panels*) and c-Ret-Y1062-phosphorylated (*arrowheads* in *lower panels*) PCs from 5, 8, 12, and 15-day-old WT mice. *B*, percentage (mean ± SD, n = 5) of Y1062-phosphorylated PCs in WT mice. The results of 2–3 serial sections from five mice are shown with dot plots. *C*, c-Ret-expressing (*arrows* in *left panels*) and c-Ret-Y1062-phosphorylated (*arrows* in *right panels*) PCs from 14-day-old WT- (*upper panels*) and YF/YF-mice (*lower panels*). *D*, percentage (mean ± SD, n = 4) of Y1062-phosphorylated PCs in 14-day-old WT mice (WT) and *c-Ret*-KI^YF/YF^-mice (YF/YF). Scale bars: 20 μm. The results of four serial sections from four mice are shown with dot plots. *E*, retention times (seconds, mean ± SD) of YF/YF-mice (*open circles*, n = 7) and littermate WT mice (*black circles*, n = 7) on the rotarod (at 25 rpm) were recorded. *F*, footprint analysis. *G*, quantification of stride length (cm, mean ± SD) of YF/YF-mice (n = 5) and littermate WT mice (n = 5). The results of three trials from five mice are shown with dot plots. Significant difference (∗∗*p* < 0.01; ∗*p* < 0.05) from the control was analyzed by the paired *t* test (*B*), the *Mann–Whitney U* test (*E*) and the unpaired *t* test (*D*, *G*).

c-Ret protein was constantly detectable in PCs until postnatal day (P) 15, while phosphorylated Y1062 in c-Ret was detectable in PCs after P8 and gradually increased until P15 in wild-type (WT) mice, several days before WT mice had complete cerebellar development (Fig. 1, *A* and *B*). The number of Y1062-phosphorylated PCs was undetectably small in *c-Ret*-KI^YF/YF^-mice even on P14 compared with that in WT mice on P14 (Fig. 1, *C* and *D*). We next analyzed the coordinate performance of *c-Ret*-KI^YF/YF^-mice. *c-Ret*-KI^YF/YF^-mice were ataxic with impaired performance on the rotarod and a waddling gait pattern on P21 (Fig. 1, *E*–*G*).

### Cerebellar hypoplasia in homozygous c-Ret-KI^YF/YF^-mice

**Figure 2:**
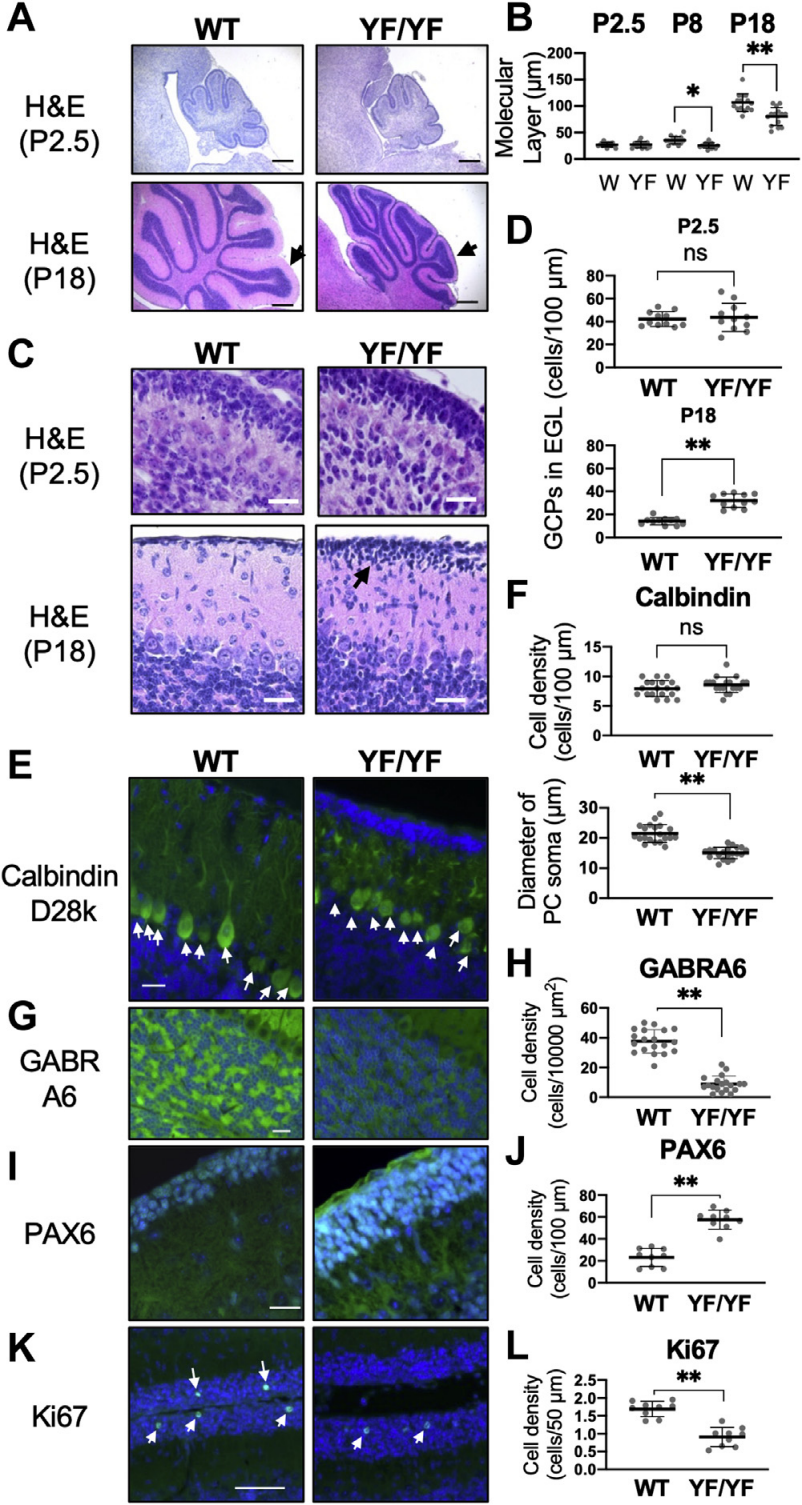
**Cerebellar hypoplasia in *c-Ret* knock-in mice.***A–D*, histology of the cerebellum from 2.5-day-old (P2.5, *upper panels* in *A*, *C*) and 18-day-old (P18, *lower panels* in *A*, *C*) YF/YF-mice (*right panels* in *A*, *C*) and WT mice (*left panels* in *A*, *C*). *B*, thickness of the molecular layer (μm, mean ± SD, n = 5) in the posterior fissure (indicated by arrows in lower panels in *A*) from YF/YF-mice (YF) and WT mice (W) at P2.5, P8 and P18. The results of four serial sections from five mice are shown with dot plots. *C* and *D*, numbers (cells per 100 μm, mean ± SD) of GCPs in the EGL from 2.5-day-old (P2.5, upper panels in *C*) and 18-day-old (P18, lower panels in *C*) YF/YF-mice (*white bars*, n = 5) and WT mice (*black bars*, n = 5). The results of three serial sections from five mice are shown with *dot plots*. *E* and *F*, numbers of cells (cell number per 100 μm, mean ± SD, n = 5) positive for calbindin D28k (*upper graph*) and diameters of PC somas (μm, mean ± SD, n = 5) in 21-day-old YF/YF-mice and WT mice obtained from immunohistochemistry are shown in *E*. *Arrows* in *E* indicate positive cells. The results of four serial sections from five mice are shown with *dot plots*. *G*–*L*, numbers of positive cells for GABRA6 (*G*, *H*, cells per 10,000 μm^2^, mean ± SD, n = 5 on P13), PAX6 (*I*, *J*, cells per 100 μm, mean ± SD, n = 3 on P11) and Ki67 (*K*, *L*, cells per 50 μm, mean ± SD, n = 3 on P8) in YF/YF-mice and WT mice obtained from immunohistochemistry are shown. *Arrows* in *K* indicate positive cells. The results of three serial sections from 3 to 5 mice are shown with dot plots. Scale bars: 20 μm (*C*, *E*, *G*, *I*, *K*) and 200 μm (*A*). Significant difference (∗∗*p* < 0.01; ∗*p* < 0.05) from the control was analyzed by the unpaired *t* test.

Eight- and 18-day-old *c-Ret*-KI^YF/YF^-mice showed impaired morphology of the cerebellum with less thickness of the molecular layer than that in WT mice, whereas the morphology of the cerebellum in WT mice and that in *c-Ret*-KI^YF/YF^-mice were comparable on P2.5 (Fig. 2, *A* and *B*). Eighteen-day-old *c-Ret*-KI^YF/YF^-mice showed a higher density of GCPs in the EGL than that in WT mice, while the density of GCPs in the EGL in WT mice and that in *c-Ret*-KI^YF/YF^-mice were comparable on P2.5 (Fig. 2, *C* and *D*). *c-Ret*-KI^YF/YF^-mice showed a decreased number of GABA A receptor alpha 6 (GABRA6)-positive matured granule cells (GCs) compared with that in WT mice on P13 (Fig. 2, *G* and *H*). On the other hand, WT mice and *c-Ret*-KI^YF/YF^-mice showed comparable numbers of PCs on P21 (Fig. 2, *E* and *F*, upper graph), while the diameters of somas of PCs in *c-Ret*-KI^YF/YF^-mice were smaller than those in WT mice (Fig. 2*F*, lower graph) and PCs in *c-Ret*-KI^YF/YF^-mice showed impaired electrophysiological parameters (Fig. S2) and dendritic outgrowth (Fig. 2*E*). The number of PAX6-positive GCPs in the EGL in *c-Ret*-KI^YF/YF^-mice was more than that in WT mice on P11 (Fig. 2, I and *J*). The number of Ki67-positive GCPs in the EGL in *c-Ret*-KI^YF/YF^-mice was less than that in WT mice on P8 (Fig. 2, *K* and *L*).

### Decreases in the number of glial fibers and Shh levels in the EGL in c-Ret-KI^YF/YF^-mice

**Figure 3:**
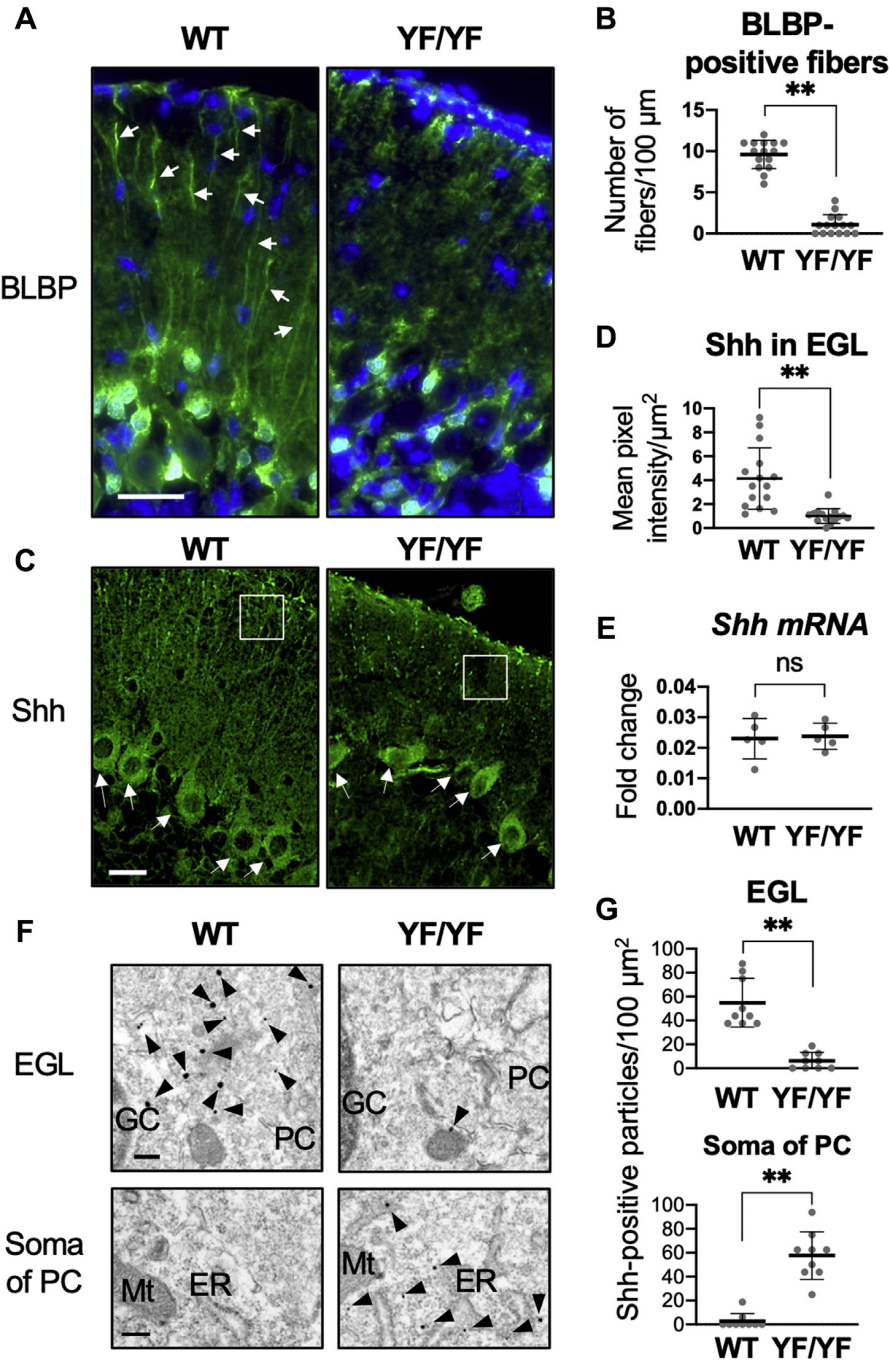
**Decreases in the number of glial fibers and Shh levels in the EGL in *c-Ret*-KI**^**YF/YF**^**-mice.***A–D*, immunohistochemical analysis of (*A*) BLBP-positive glial fibers and (*C*) Shh-positive signals from 15-day-old WT mice and YF/YF-mice. *Arrows* indicate BLBP-positive *glial fibers* (*A*) and Shh-positive PCs (*C*), respectively. *B*, number of BLBP-positive glial fibers (mean ± SD, per 100 μm) with lengths of more than 50 μm were determined in the EGL of WT mice and YF/YF-mice (n = 5, each). *D*, mean pixel intensity (mean ± SD) of Shh-positive signals determined in the EGL is indicated by squares shown in (*C*) of WT mice and YF/YF-mice (n = 5, each). *B* and *D*, the results of three serial sections from five mice are shown with dot plots. *E*, expression levels (mean ± SD) of *Shh* transcripts in the cerebellum from 17-day-old WT mice and YF/YF-mice are shown with *dot plots* (n = 5, each). *F* and *G*, immuno-electron microscopy of Shh-positive vesicles from 13-day-old littermate WT mice and YF/YF-mice (n = 3, each). *Arrowheads* indicate Shh-positive vesicles. *G*, number (mean ± SD, per 100 μm^2^) of Shh-positive particles in the EGL and somas of PCs. The results of three serial sections from three mice are shown with dot plots. Significant difference (∗∗*p* < 0.01; ∗*p* < 0.05) from the control was analyzed by the unpaired *t* test. Scale bars: 20 μm (*A*, *C*) and 500 nm (*F*). ER, endoplasmic reticulum; GC, granule cells; Mt, mitochondria; PC, Purkinje cells.

Next, glial fibers of Bergmann glia, which are essential for normal migration of GCs, in *c-Ret*-KI^YF/YF^-mice were determined to investigate the reason why *c-Ret*-KI^YF/YF^-mice had immature GCs. The number of BLBP-positive glial fibers in the EGL was significantly decreased in *c-Ret*-KI^YF/YF^-mice on P15 compared with that in WT mice (Fig. 3, *A* and *B*). The expression of *BLBP* transcripts was significantly decreased in *c-Ret*-KI^YF/YF^-mice compared with that in littermate WT mice at P17 (Fig. S3). In a previous study, the cerebellar hypoplasia of DS mice was shown to have impaired development of GCs caused by decreased Sonic hedgehog (Shh)-mediated signaling activity (20). Our results and the results of the previous study encouraged us to analyze the involvement of Shh in c-Ret-mediated cerebellar hypoplasia. Shh levels in the EGL were decreased in *c-Ret*-KI^YF/YF^-mice on P15 compared with those in WT mice (Fig. 3, *C* and *D*), while *Shh* mRNA levels were comparable in WT mice and *c-Ret*-KI^YF/YF^-mice (Fig. 3*E*). Immuno-electron microscopy further showed a decreased number of Shh-positive particles in the EGL in *c-Ret*-KI^YF/YF^-mice compared with that in WT mice (Fig. 3, *F* and *G*, upper graph). In contrast, the number of Shh-positive particles in the somas of PCs in *c-Ret*-KI^YF/YF^-mice was larger than that in WT mice (Fig. 3, *F* and *G*, lower graph). In a previous study, axonal trafficking of Shh with synaptic vesicles 2 (SV2) was shown, and it was also shown that mutant Shh caused decreased secretion as well as impaired trafficking in primary neurons (21). In this study, *c-Ret*-KI^YF/YF^-mice had decreased expression of SV2 and less colocalization of Shh with SV2 in the EGL than those in WT mice (Fig. S4).

### c-Ret-mediated cerebellar hypoplasia with immature GCs was rescued by a smoothened agonist (SAG) and by reduced expression of Patched1

**Figure 4:**
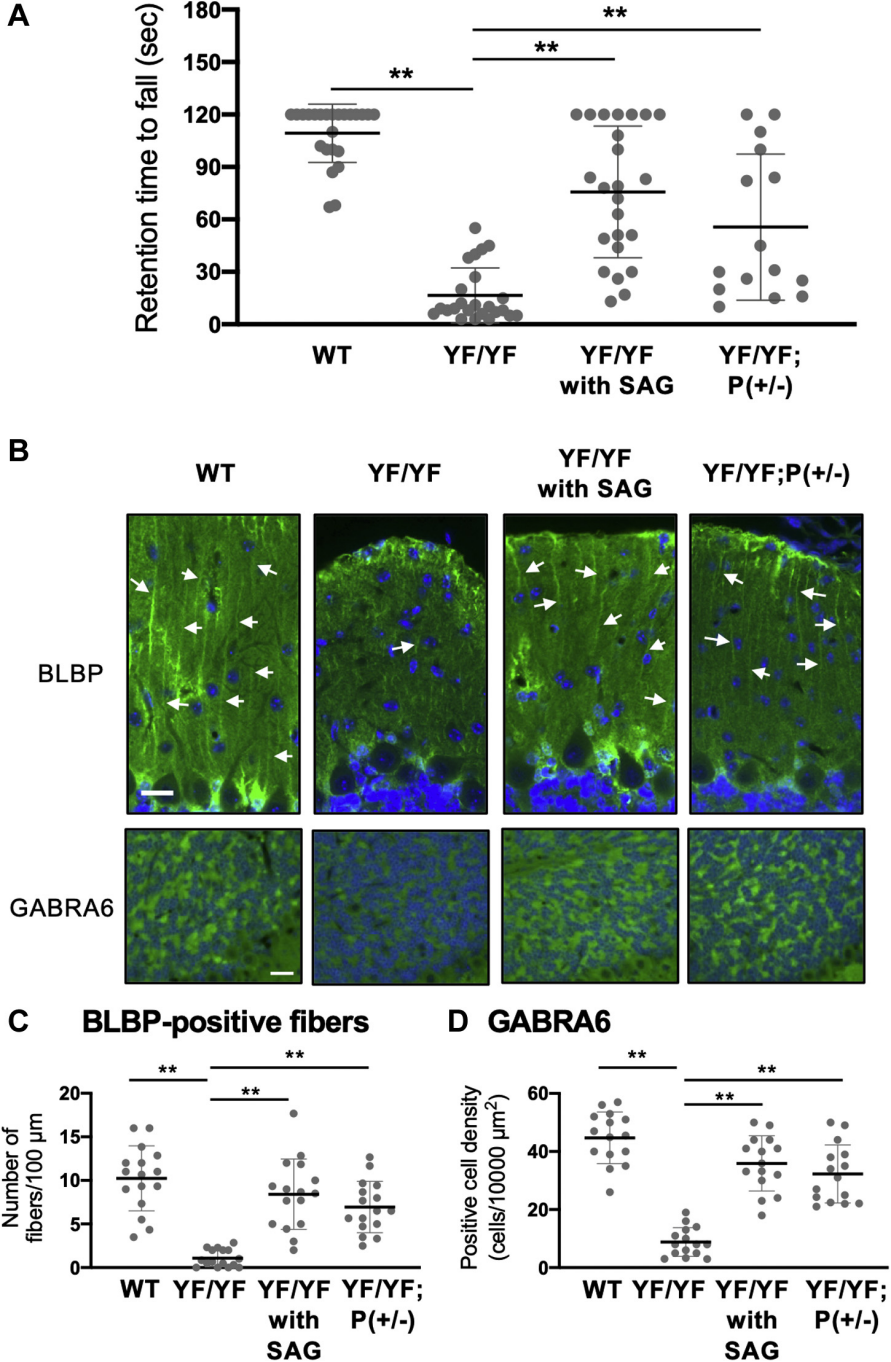
**c-Ret-mediated cerebellar hypoplasia with immature GCs was rescued by SAG treatment and by reduced expression of *Patched1*.***A*, retention times (seconds) of 21-day-old YF/YF-mice (n = 8), YF/YF-mice treated with SAG (n = 8), YF/YF;*Patched1*-KO(+/−)-mice (YF/YF;P(+/−), n = 5) and littermate WT mice (n = 8) on the rotarod (at 5 rpm) are shown. Four groups were allowed a maximum retention time of 120 s per trial. The results of triplicate measurements with 5-min intervals after having training twice are shown with *dot plots*. *B*, immunohistochemistry of 21-day-old WT mice, YF/YF-mice, YY/YF-mice treated with SAG and YF/YF;P(+/−)-mice using anti-BLBP (*upper panels*) and anti-GABRA6 (*lower panels*). Scale bars: 20 μm. *C*, number of BLBP-positive glial fibers (mean ± SD, per 100 μm) with lengths of more than 50 μm in the EGL and (*D*) GABRA6-positive cell density (mean ± SD, per 10,000 μm^2^) in WT mice, YF/YF-mice, YY/YF-mice treated with SAG and YF/YF;P(+/−)-mice (n = 5, each). The results of three serial sections from five mice are shown with dot plots. Significant difference (∗∗*p* < 0.01) was analyzed by the Steel–Dwass test.

We tried to rescue the cerebellar hypoplasia in *c-Ret*-KI^YF/YF^-mice with subcutaneous injection of SAG, a smoothened agonist (20) in order to verify the involvement of Shh in the ataxic phenotype and the decreased number of matured GCs in *c-Ret*-KI^YF/YF^-mice. The ataxic phenotype in *c-Ret*-KI^YF/YF^-mice was partially rescued by SAG (Fig. 4*A*). Decreased numbers of BLBP-positive glial fibers and GABRA6-positive mature GCs in *c-Ret*-KI^YF/YF^-mice were significantly rescued in *c-Ret*-KI^YF/YF^-mice treated with SAG (Fig. 4, *B*–*D*). We finally crossed *c-Ret*-KI^YF/+^-mice and *Patched1* knockout mice to examine whether c-Ret-mediated cerebellar hypoplasia is rescued by decreased expression of *Patched1*, a receptor for Shh, and a negative regulator for Shh-mediating signaling (22, 23). The ataxic phenotype and the decreased numbers of BLBP-positive glial fibers and matured GCs in *c-Ret*-KI^YF/YF^-mice were also rescued in *c-Ret*-KI^YF/YF^;*Patched1*-KO(+/−)-mice (Fig. 4, *A*–*D*). On the other hand, the c-Ret-mediated megacolon phenotype of HSCR was not rescued by reduced expression of *Patched1* at least in this experimental condition (Fig. S5), although we have not examined the effect of SAG on HSCR.

## Discussion

This study demonstrated for the first time that impaired phosphorylation of Y1062 in c-Ret caused cerebellar hypoplasia in *c-Ret*-KI^YF/YF^-mice, although the sample sizes in this study were limited. Impaired development of GCs was shown to be involved in cerebellar hypoplasia in *c-Ret*-KI^YF/YF^-mice at P18. In contrast, the numbers of GCPs were comparable in *c-Ret*-KI^YF/YF^-mice and WT mice on P2.5. Our results also demonstrated that Y1062-phosphorylation in c-Ret of PCs was undetectable on P5 but gradually increased after P8 at least until P15. Thus, these results suggest that GCs even in *c-Ret*-KI^YF/YF^-mice developed normally at least until P2.5, when Y1062-phoshorylation in c-Ret of PCs from WT mice was undetectable, but that GCPs were accumulated in the EGL in *c-Ret*-KI^YF/YF^-mice on P8-18, when the level of Y1062-phoshorylation in c-Ret of PCs from WT mice was high. In previous studies *in vitro*, Y1062-phosphorylation in c-Ret was shown to be crucial for neurite outgrowth (24, 25, 26), although there is no information about the influence on PCs *in vivo*. In this study, *c-Ret*-KI^YF/YF^-mice at P18 showed decreased soma size and shorter outgrowth of neurites in PCs than those in WT mice, suggesting that impaired phosphorylation of Y1062 in c-Ret caused impaired morphology in PCs. Our results partially correspond to the results of previous studies showing decreased soma size of dorsal root ganglion neurons in *Ret-floxed;Wnt1-Cre* mice (27) and impaired development of GCs as well as PCs in DS model mice (Ts65Dn) (20) and *staggerer* (*sg/sg*) mice (28, 29). Also, our results are similar to the results of a study showing impairment of cerebellar motor learning in mice with tissue-specific deletion of *c-Ret* in molecular layer interneurons in the cerebellum (30). Thus, this study suggests that *c-Ret*-KI^YF/YF^-mice have phenotypes of HSCR-DS including cerebellar hypoplasia.

Our results showed impairment of BLBP-positive glial fibers of Bergmann glia, which are known to be required for migration of GCs, in *c-Ret*-KI^YF/YF^-mice. We also determined the expression of *BLBP* transcripts in the cerebellum in *c-Ret*-KI^YF/YF^-mice, since the expression of BLBP is known to be crucial for the development of glial fibers of Bergmann glia (31). The *BLBP* level was decreased in *c-Ret*-KI^YF/YF^-mice compared with that in littermate WT mice. The finding is similar to the results of a previous study showing decreased expression of *BLBP* transcripts (also called *Fabp7*) in the ENS in *Ret*-knockout mice at embryonic day 14 (32). In a previous study, impaired migration of GCs was shown to cause immature formation of parallel fiber-PC synapses in cerebellar hypoplasia (29). Therefore, our results suggest the possibility that impaired development of glial fibers by decreased expression of *BLBP* resulted in the formation of immature synapses due to impaired migration of GCs in *c-Ret*-KI^YF/YF^-mice.

Our results and the results of a previous study (11) showed that c-Ret is expressed in PCs but not in GCs or Bergmann glia from 14-day-old WT mice. Therefore, our results raised the possibility that decreased phosphorylation of c-Ret Y1062 in PCs causes impaired cross talk *via* a soluble factor among PCs, Bergman glia, and GCs, resulting in cerebellar hypoplasia. In this study, we focused on the involvement of the mitogenic factor Shh, since previous studies showed that (i) Shh induced the expression of *BLBP* (33) and (ii) Shh secreted from PCs is one of the key factors for cerebellar development by facilitating expansion of glial fibers of Bergmann glia (34) and migration of GCs along glial fibers (35, 36) and proliferating GCPs (35). In this study, *c-Ret*-KI^YF/YF^-mice at P8 had a decreased number of Ki67-positive GPCs in the EGL compared with that in WT mice, suggesting that proliferation of GCPs in the EGL was also affected in *c-Ret*-KI^YF/YF^-mice. Thus, our results suggest the possibilities that migration and proliferation of GCPs are impaired in *c-Ret*-KI^YF/YF^-mice. There is also the possibility that the impaired Shh from PCs affects the final maturation of GCs after migration, since it seems that the cell number of GCs in the IGL in *c-Ret*-KI^YF/YF^-mice was comparable to that in WT mice, although the expression level of GABRA6 in *c-Ret*-KI^YF/YF^-mice was less than that in WT mice. In a previous study, the expression level of GABRA6 in cerebellar GCs was shown to be regulated by nuclear factor I (NFI) proteins (37). Shh has been shown to increase the expression of NFI in dental mesenchyme (38). Thus, it is likely that impaired Shh from PCs decreases the expression of GABRA6 *via* NFI in GCs in *c-Ret*-KI^YF/YF^-mice. GABRA6 has been shown to play an important role for mediating tonic conductance *via* a voltage-independent K^+^ conductance in mature GCs in the cerebellum (39). Therefore, there is a possibility that cerebellar hypoplasia in *c-Ret*-KI^YF/YF^-mice involves impaired function of GCs.

Our results demonstrated that Shh-positive vesicles in the EGL in *c-Ret*-KI^YF/YF^-mice were decreased compared with those in WT mice. In contrast, Shh-positive vesicles in the somas of PCs in in *c-Ret*-KI^YF/YF^-mice were increased compared with those in WT mice, although the transcriptional levels of Shh in the cerebellum were comparable in WT mice and *c-Ret*-KI^YF/YF^-mice. Our results are partially similar to the results of a study showing impaired development of GCs with decreased activity of Shh-mediated signaling in DS mice (20). In this study, *c-Ret*-KI^YF/YF^-mice showed decreased expression of SV2, which was shown to be required for axonal trafficking of Shh (21) and less colocalization of Shh with SV2 in the EGL than that in WT mice. A previous study showed positive expression of SV2 in medullary thyroid carcinoma caused by a gain-of-function mutation in *RET* (40). Therefore, it is likely that *c-Ret*-KI^YF/YF^-mice, which are mice with a loss-of-function mutation of *c-Ret*, have decreased expression of SV2. Thus, it is possible that impaired phosphorylation of Y1062 in c-Ret causes impairments of neurite outgrowth and SV2-mediated Shh trafficking in PCs, resulting in hypoplasia of GCs and glial fibers with decreased expression of *BLBP*. In a previous study, Sortilin, a multifunctional sorting receptor, was shown to regulate Shh trafficking (41). Therefore, it is possible that Shh trafficking is mediated by not only SV2-mediated processes but also other processes. Further study is needed to investigate whether c-Ret-mediated signaling correlates with Shh trafficking mediated by other processes.

In a previous study, subcutaneous injection of a smoothened agonist (SAG) rescued the impaired development of GCs in the cerebellum in DS mice (20). Our results showed the rescue effect of subcutaneous injection of SAG after birth on *c-Ret*-mediated cerebellar hypoplasia including a decreased number of mature GCs as well as glial fibers, while the glial fibers appeared thicker and swollen compared with those in WT mice or *c-Ret*-KI^YF/YF^;*Patched1*-KO(+/-)-mice. This study further demonstrated that cerebellar hypoplasia in *c-Ret*-KI^YF/YF^-mice was rescued by reduced expression of *Patched1*, a negative regulator for Shh-mediating signaling (22, 23). Thus, our results suggest that *c-Ret*-KI^YF/YF^-mice have cerebellar hypoplasia *via* Shh-mediated impairments of GCs and glial fibers. Further study is needed to examine the rescue effects of smoothened agonists on HSCR.

In conclusion, this study demonstrated cerebellar hypoplasia in *c-Ret*-KI^YF/YF^-mice. In a previous study, severe HSCR with total colonic aganglionosis of the ENS was shown in *c-Ret*-KI^YF/YF^-mice (3). An association of *RET* mutations with HSCR-DS in patients with total colonic aganglionosis was also shown (14). However, there is very limited information about cerebellar hypoplasia in HSCR-DS patients caused by *RET* mutations. Therefore, further studies are needed to investigate *RET*-mediated cerebellar hypoplasia in patients and to develop new therapeutic strategies targeting *RET*-related molecules against cerebellar hypoplasia.

## Experimental procedures

### Mice

*c-Ret*-KI^YF/YF^ mice (YF/YF) were previously reported (3, 19). There have been no reports of cerebellar hypoplasia in this strain. In this study, *c-Ret*-KI^YF/YF^;*Patched1*-KO(+/−) mice (YF/YF;P-KO[+/−]) were newly established by crossing *c-Ret*-KI^YF/+^ mice with *Patched1*-KO(+/−) mice (23). All of the experiments in this study were approved by the Institutional Animal Care and Use Committee in Nagoya University (approval number: 20238) and the Institutional Recombinant DNA Experiment Committee in Nagoya University (approval number: 20-24) and followed the Japanese Government Regulations for Animal Experiments.

### Behavior analyses

Cerebellar hypoplasia in mice was assessed by performance on a rotarod analysis with a rotating rod treadmill (Ugo Basile; Stoelting Co) and by a footprint test as previously reported (42, 43, 44). For the rotarod test, the performance of mice at P21 on the rod until a maximum retention time of 120 s per trial was assessed. The latency (seconds) when each mouse was not able to keep walking on the rotating rod was recorded. Six successive trials on the rotating rod with 5-min intervals were performed and the results are shown in Figure 1*E*. For the results shown in Figure 4*A*, three tests on with 5-min intervals were performed after training twice on the rotating rod. For the footprint test, the front paws and back paws of mice were painted red and blue, respectively. The mice were gently placed on a sheet of white paper in a 14 × 44 cm box. The length between the back edges of paw prints on the same side was determined as stride length.

### Morphological analysis by light microscopy

After perfusion fixation with Bouin's solution, cerebelli from 0.5 to 21-day-old mice were immersed in the same solution overnight or for 1 day. Sagittal sections of 6 μm in thickness were used for morphological analyses. Hematoxylin-eosin (H&E) staining was performed with paraffin sections. Immunostaining with antibodies against c-Ret (1:150; Immuno Biological Laboratories), phosphorylated c-Ret Y1062 (1:50; Abcam), and Shh (1:100; Santa Cruz, H-160) diluted with Can Get Signal immunostaining solution (TOYOBO) was performed with paraffin and frozen sections (19, 45). Immunostaining with antibodies against GABRA6 (1:1000; Chemicon), calbindin D28k (1:150; Chemicon), BLBP (1:200, Abcam), PAX6 (1:500, Abcam), and Ki67 (1:500, Abcam) was performed with paraffin sections. The VECTASTAIN Elite ABC kit (Vector), Envision kit/HRP (diaminobenzidine; DAB) (DAKO) with counterstaining of hematoxylin and Alexa Fluor 488-labeled donkey anti-rabbit IgG (1:1000, Invitrogen) with counterstaining of 4′, 6-diamidino-2-phenylindole (DAPI) were used. We validated the primary antibodies used in this study with no positive signals in the specimens processed under the same staining condition except for incubation without primary antibodies.

### Morphological evaluations

Determination of the thickness of the molecular layer in the posterior fissure and the density of GCPs in the EGL with H&E staining basically followed the previous method (46). In brief, boxes of 100 μm in length were randomly placed anterior to the primary fissure. The number of GCPs in each box was determined on three sections from the most medial 100 μm of each mouse. The results of 3–4 serial sections from five mice of each genotype were used for the evaluation. For immunohistochemical determination of cells or glial fibers stained with antibodies, the software program WinROOF ver. 6.5 (Mitani Corp) was used as previously reported (19, 44). The percentage of phosphorylation of Y1062 in c-Ret in PCs was calculated by dividing the number of phosphorylated c-Ret-positive PCs by the number of c-Ret-positive PCs in the precentral and preculminate fissures and lobes II and III from two to four serial sections of 4–5 mice for each mouse strain. Examples of Y1062-phosphorylated PCs recognized by the software are shown in Figure S6. The number of positive cells or glial fibers was determined in boxes of 50 μm or 100 μm in length randomly placed in the Purkinje layer (stained by anti-calbindin D28k), in the IGL (stained by anti-GABRA6), and in the EGL (stained by anti-BLBP, anti-PAX6 and anti-Ki67) anterior to the primary fissure from three to four serial sections of 3–5 mice for each mouse strain. Mean pixel intensity of Shh in boxes (20 μm × 20 μm) placed randomly in the EGL was analyzed with WinROOF.

### Immuno-electron microscopy

Immuno-EM was basically performed by the previously described method (47). After perfusion fixation with 2% (vol/vol) paraformaldehyde and 2% (vol/vol) glutaraldehyde (GA) in 0.1 M phosphate buffer (PB), brain tissues were further postfixed for 1 h at 4 °C. Then cerebellum tissues of 40 μm in thickness were prepared with a vibratome (Leica). After pretreatment with 0.05% Triton-X100 in PBS for 20 min at 4 °C, the tissues were incubated in 5% normal goat serum for 30 min at 4 °C. The tissues were incubated with anti-Shh (1:10; Santa Cruz, H-160) in PBS overnight at 4 °C, followed by incubation with anti-rabbit IgG labeled with 1.4-nm gold particles (1:50) in PBS overnight at 4 °C. After washing, the labeled tissues were postfixed in 2% GA in 0.1 M PB at 4 °C overnight. After washing again, the tissues were gradually dehydrated with a stepped series of ethanol and eventually embedded in epoxy resin (Quetol-812; Nisshin EM). An electron microscope at 80 kV (JEM-1400Plus; JEOL) was used to observe ultrathin sections of 80 nm in thickness. Positive particles per one view (4 μm × 4 μm) in the EGL and somas of PCs from three mice of each strain were determined.

### Real-time PCR

Real-time PCR was performed by a method described previously (48). The transcriptional levels of *Shh* were determined by quantitative RT-PCR (real-time PCR) with SYBR green (Roche, 04913850001). The levels were normalized with *Hprt* expression. Sequences of primers for *Shh* were 5′-CCAATTACAACCCCGACATC-3′ and 5′-GCCACTGGTTCATCACAGAG-3′, and those for *Hprt* were 5′-CTTTGCTGACCTGCTGGATT-3′ and 5′-TATGTCCCCCGTTGACTGAT-3′.

### Administration of SAG

We partially followed the method used in a previous study (20). A smoothened agonist, SAG_1.3_ (N-Methyl-Nʹ-(3-pyridinylbenzyl)-Nʹ-(3-chlorobenzo[b]thiophene-2-carbonyl)-1,4-diaminocyclohexane; EMD Chemicals, single dose at 20 μg/g body weight), was subcutaneously injected into YF/YF mice three times at P1.5, P2.5, and P3.

### Statistical analysis

A significant difference between two groups for parametric data was determined by the two-tailed unpaired *t* test. For determination of significant differences among more than three groups for parametric data, one-way ANOVA followed by Tukey's post hoc multiple comparison test was used. For nonparametric data, the *Mann*–*Whitney U* test and the Steel–Dwass test with the alpha level set to 0.05 were used as previously reported (44). Values of *p* < 0.05 were considered to be statistically significant. All statistical analyses were performed using Prism 9 (GraphPad).

## Data availability

All data are contained within the article.

## Supporting information

This article contains supporting information.

## Conflict of interest

The authors declare that they have no conflicts of interest with the contents of this article.
