# Supplementary material for: Loss-of-function mutation of c-Ret causes cerebellar hypoplasia in mice with Hirschsprung disease and Down's syndrome
Source: J Biol Chem. 2021 Feb 6;296:100389. doi: 10.1016/j.jbc.2021.100389 (PMC7950328; doi:10.1016/j.jbc.2021.100389)
Supplement: Supporting information [file mmc1.pdf]

Loss-of-function mutation of c-Ret causes cerebellar hypoplasia in mice with Hirschsprung disease and Down's syndrome

Nobutaka Ohgami, Akira Iizuka, Hirokazu Hirai, Ichiro Yajima, Machiko Iida, Atsuyoshi Shimada, Toyonori Tsuzuki, Mayumi Jijiwa, Naoya Asai, Masahide Takahashi and Masashi Kato

Supplementary Information

SI materials and methods

Electrophysiological analysis. Electrophysiological analysis of Purkinje cells (PCs) with parasagittal cerebellar slices (200–250 μm in thickness) from 21-day-old mice basically followed the procedure used in a previous study (49).

49. Mitsumura, K., Hosoi, N., Furuya, N., Hirai, H. (2011) Disruption of metabotropic glutamate receptor signalling is a major defect at cerebellar parallel fibre-Purkinje cell synapses in staggerer mutant mice. *J. Physiol.* **589**, 3191-3209

Supplementary Figures

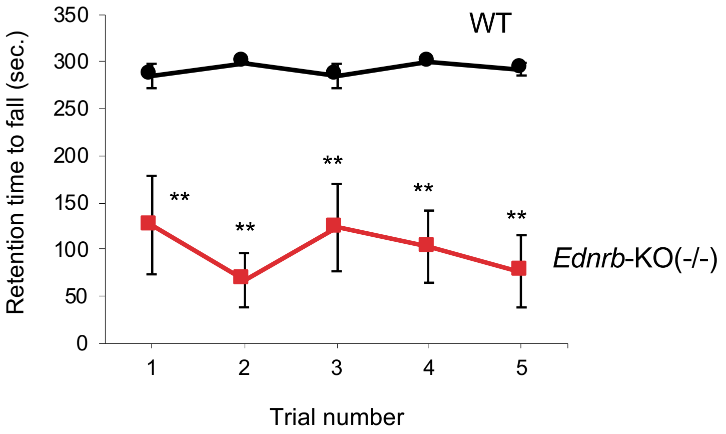

Figure S1. Rotarod analysis of 21-day-old *Ednrb-knock out* (-/-) mice. Retention times (seconds) on the rotarod (at 25 rpm) were recorded. Twenty-one-day-old *Ednrb-knock out* (-/-) mice (n=4) (Ida-Eto, et al., 2011) and littermate wild-type mice (WT, n=4) were allowed a maximum retention time of 300 seconds per trial. Significant difference (**, $p < 0.01$) from the control was analyzed by the unpaired t-test.

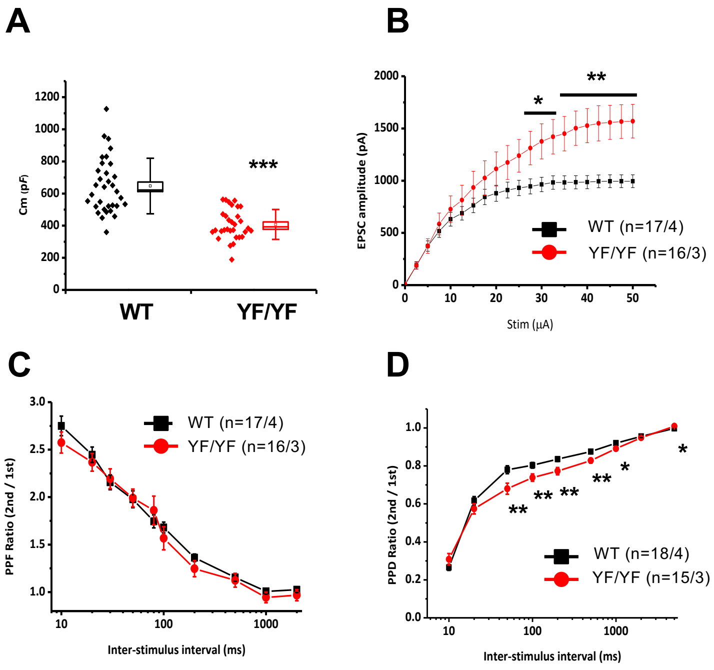

Figure S2. Electrophysiological analysis of PCs in YF/YF-mice and their WT littermates. (**A**) Significant decrease in membrane capacitance (Cm) of PCs from YF/YF-mice, suggesting impaired dendritic differentiation of PCs in YF/YF-mice. Plots in the boxes are averages of data. Upper and lower edges of the boxes and whiskers show SE and SD, respectively. (**B**) Input-output curves showing amplitude of EPSCs elicited in PCs in response to increasing electrical stimulation to parallel fibers (PFs). PF stimulation over 27.5 μA produced a significantly larger EPSC response in YF/YF-mice than in WT mice. **(C)** Paired pulse facilitation of PF-PC EPSCs. (**D**) Paired pulse depression (PPD) ratio was significantly decreased in YF/YF-mice compared with that in WT mice. The number of cells (n) and number of animals examined are shown in parentheses (PCs/animals). Significant difference (***, $p < 0.001$; **, $p < 0.01$; *, $p < 0.05$) from the control was analyzed by the Mann-Whitney *U* test.

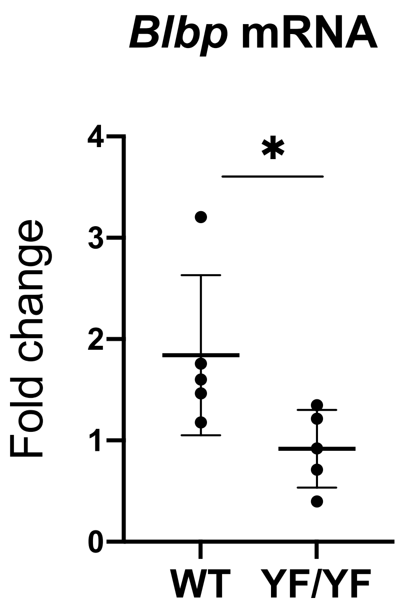

Figure S3. Decreased expression of *BLBP* in the cerebellum in *c-Ret*-KI$^{YF/YF}$-mice. Expression levels (mean ± SD) of *BLBP* transcripts in the cerebellum from 17-day-old WT mice and YF/YF-mice (n=5, each) were determined by real-time PCR with SYBR green. The levels were normalized with *Hprt* expression. Sequences of primers for *BLBP* were 5'-GAAGTGGGATGGCAAAGAAA-3' and 5'-GGGACTCCAGGAAACCAAGT-3'. Significant difference (*, $p < 0.05$) from the control was analyzed by the unpaired t-test.

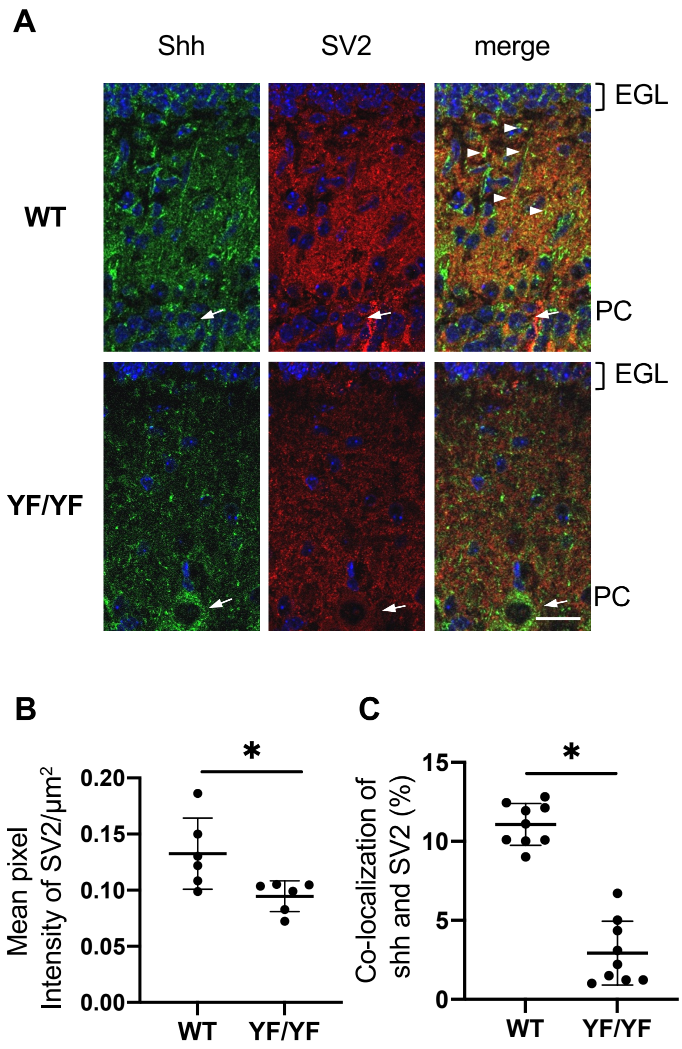

Figure S4. Decreased co-localization of Shh and SV2 in the EGL in *c-Ret*-KI$^{YF/YF}$-mice. (**A**) Immunohistochemical analysis of Shh (green; rabbit anti-Shh antibodies; Santa Cruz; 1:100) and SV2 (red; goat anti-SV2 antibodies, Santa Cruz; 1:50) with frozen sections (6 μm in thickness) of the cerebellum from 13-day-old WT mice and YF/YF mice (n=3, each). Counterstaining with DAPI. Dot-patterns of SV2 signals were observed as shown in a previous study (Meehan et al., *J. Neurophysiol.* **106**, 1227-1239, 2011). Arrows indicate PC and arrowheads indicate typical examples of overlapped signals of Shh and SV2. Scale bar: 20 μm. (**B**) Mean pixel intensity (mean ± SD) of SV2-positive signals determined in the EGL of WT mice and YF/YF-mice. (**C**) Co-localization of Shh and SV2 in the EGL (%, mean ± SD) was analyzed with an LSM880 confocal microscope and ZEN 2009 software (Carl Zeiss). The results of (**B**) 2 serial sections and (**C**) 3 serial sections from 3 mice per each group are shown with dot plots. Significant difference (*, $p < 0.05$) from the control was analyzed by the unpaired t-test.

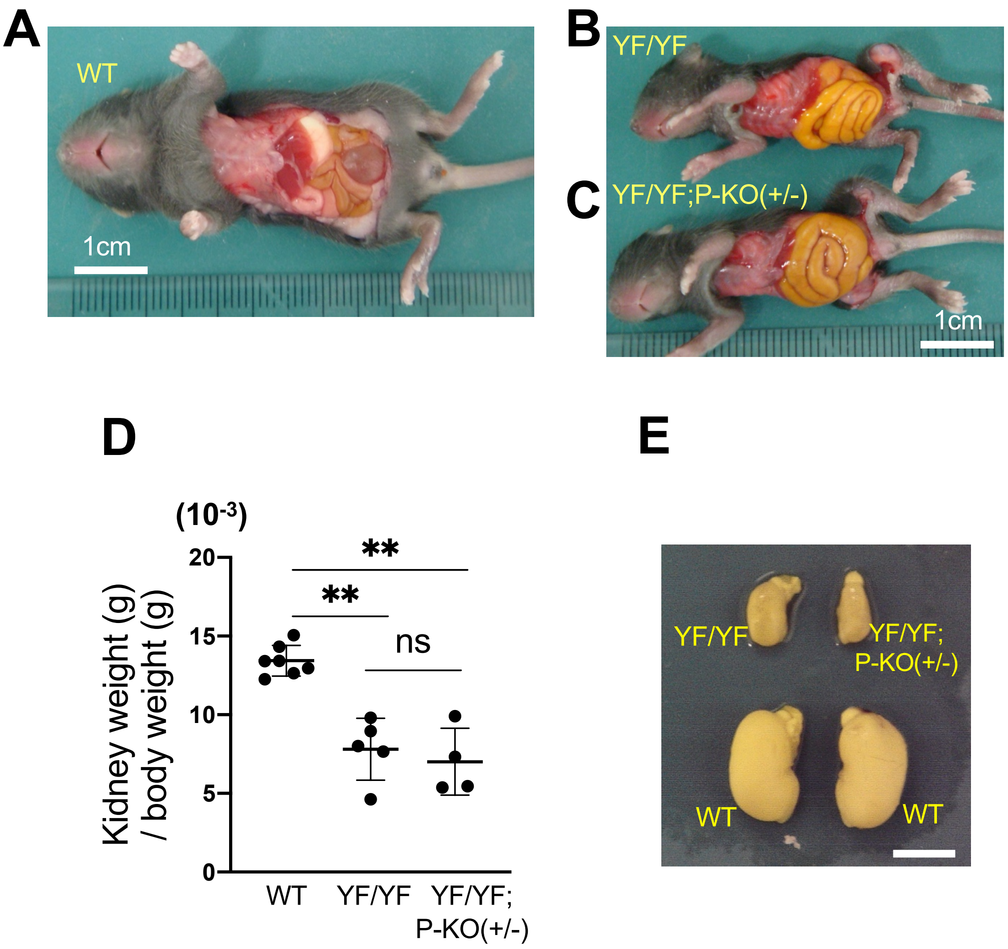

Figure S5. Megacolon and impaired development of the kidney in *c-Ret*-KI$^{YF/YF}$-mice were not rescued by reduced expression of *Patched1*. (**A-C**) Macroscopic abdominal and intestinal observations. Distended abdomen and dilated small intestine in a *c-Ret*-KI$^{YF/YF}$-mouse (**B**) were not rescued by reduced expression of Patched1 [*c-Ret*-KI$^{YF/YF}$;*Patched1*-knockout(+/-) mouse; YF/YF;P-KO(+/-)] (**C**), while a WT mouse (**A**) showed normal morphology of the abdomen. These pictures are mice having typical phenotypes. The graduation in the ruler in (**A-C**) is one millimeter. Scale bars: 1 cm. (**D**) Reduced kidney weight-to-body weight (mean ± SD) in *c-Ret*-KI$^{YF/YF}$-mice (n=5) was not restored by reduced expression of Patched1 [*c-Ret*-KI$^{YF/YF}$;*Patched1*-knockout(+/-)-mice; YF/YF;P-KO(+/-)] (n=4) at P21, while WT mice showed normal development of the kidney (n=7). Significant difference (**, $p < 0.01$; not significance, n.s.) was analyzed by the Tukey test. (**E**) Macroscopic morphology of the kidney in each genotype is shown. Scale bar: 2.5 mm.

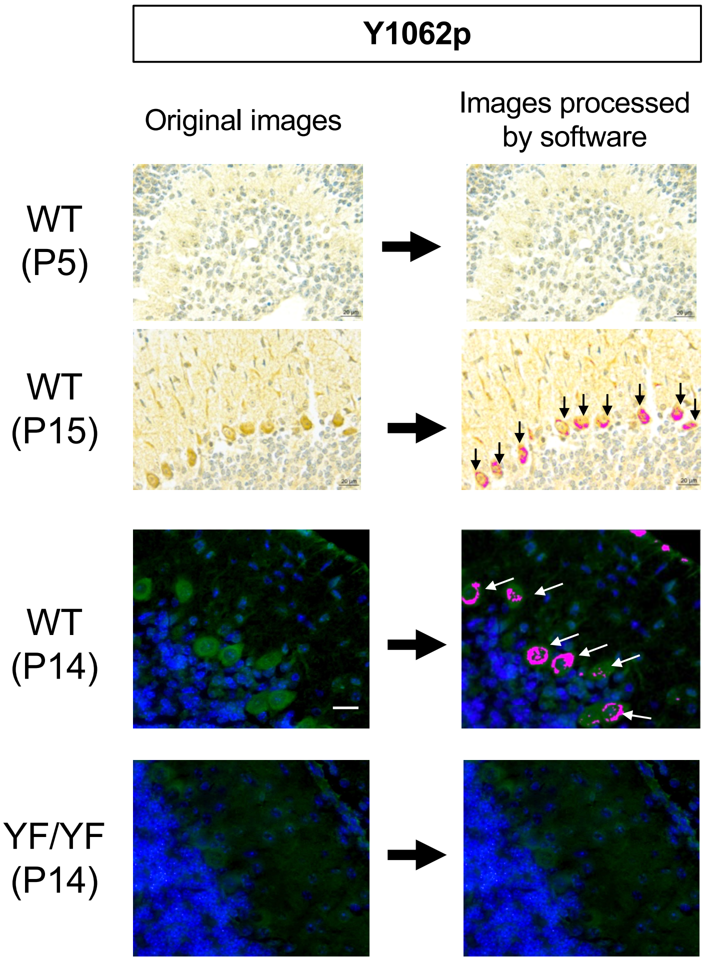

Figure S6. Purkinje cells (PCs), which were stained by anti-c-Ret Y1062p, recognized by image processing software. Examples (right panels) used for semi-quantitative analyses are shown. The original pictures (left panels) are shown in Figure 1A, C. The software program WinROOF (Ver. 6.5) was used to objectively recognize positive PCs highlighted by pink (right panels, arrows) that were stained by anti-c-Ret Y1062p [left panels, brown color of (diaminobenzidine; DAB) in WT at postnatal day (P) 15 but not P5 and green fluorescence of Alexa-488 in WT but not YF/YF at P14] in order to count the number of positive PCs. The same thresholds were consistently used for the evaluation of images visualized by DAB and Alexa-488, although different thresholds were used for DAB and Alexa-488, respectively. PCs without pink were not counted as positive PCs. Scale bars: 20 μm.
